# Supplementary material for: Preoperative Biopsychosocial Assessment and Length of Stay in Orthopaedic Surgery Admissions of Youth with Cerebral Palsy
Source: Behav Sci (Basel). 2023 May 6;13(5):383. doi: 10.3390/bs13050383 (PMC10215593; doi:10.3390/bs13050383)
Supplement: Supplementary file 1 [file behavsci-13-00383-s001.zip › behavsci-2338555-supplementary.pdf]

NEMOURS CHILDREN'S HEALTH

100122

Patient Name:
MRN:
DOB:

SOCIAL WORK BIOPSYCHOSOCIAL ASSESSMENT (Downtime)

Patient Gender Identify: ____
Family's preferred language in the Health care setting: ___________________
I have reviewed the interdisciplinary progress note today: ☐Yes ☐No
Location of consult: ☐Inpatient ☐Emergency Department (ED) ☐Ambulatory

PURPOSE OF CONSULT

☐ED behavioral health screen
☐New diagnosis
☐Support/coping
☐Guardianship/consents
☐Biopsychosocial assessment
☐Psychiatric crisis
☐Concerns with medical compliance
☐Child maltreatment
☐Resources (transportation, car seat, food assistance, referrals, housing, financial)
☐Hospitalization greater than 7 days
☐Other: ______________________________________

Family's Goals for Social Work (SW) Consult: ___________________________________

FAMILY SYSTEM

1. Family/household composition, structure and roles (including siblings): _____________
2. Legal Guardian(s): ________________________________
3. Medical decisionmakers for patient: ____________________________
4. Caregiver employment: __________________________
5. Spiritual/religious/moral tradition: ____

FAMILY & COMMUNITY SUPPORTS

☐Extended family
- ________________________________

☐Friends
☐Faith community
☐In-Home health care (nursing, home infusion therapies; hospice)
- Company name: ____________________
- Services received: _______________________
- Contact person: __________________________
- Phone number: ___________________________

☐Community-based supports
☐Ronald McDonald House (RMH)
☐Disease-related services/agency
- Please state: ________________________________

☐Outpatient Physical Therapy (PT)/Occupational Therapy (OT)/Speech
- Name of provider: ______________
- Contact information: ________________________

☐Community non-profit organization

SOCIAL WORK BIOPSYCHOSOCIAL ASSESSMENT (Downtime)

- ☐Community health worker, community social worker, case manager or service coordinator
  - Name of provider: ___________________________
  - Contact information: ___________________________
- ☐Support group: __________________________

☐Mental health/substance abuse services
- Provider name: _____________
- Phone number: _____________

☐Primary care provider
- Name: ______________________
- Phone number: ________________________________

☐Pharmacy
- Name: _________________
- Address: _______________________
- Phone number: _______________________

☐Durable Medical Equipment
- Company name: ________________________
- Equipment provided: _____________________
- Contact person: _________________________
- Phone number: _________________________
- Fax number: ___________________________

☐Transportation
- ☐Personal vehicle
- ☐Relies on friends/family
- ☐Relies on insurance provided transportation
  - Company name: ___________________
  - Phone number: ___________________
- ☐Other: _______________________________

☐Government Benefits
- ☐Supplemental Security Income (SSI)
- ☐Temporary Assistance for Needy Families (TANF)
- ☐Supplemental Nutrition Assistance Program (SNAP)
- ☐Women, Infants and Children (WIC)
  - Office location: ______________________
  - Phone number: _____________________
  - Fax number: ________________________
- ☐Mental Health/Intellectual Developmental Disability (MH/IDD)

☐Vocational/rehabilitation services

☐Child protective services

☐Juvenile justice services

☐School/employer

SOCIAL WORK BIOPSYCHOSOCIAL ASSESSMENT (Downtime)

- Name: ________________
- Grade level: ________________
- Contact person: ______________
- Phone number: _________________
- Individualized Educational Plan (IEP)/504: ___________________

☐Support
☐Other: _________________________________

STRENGTHS OF PATIENT AND FAMILY

- Client/caregiver-identified strengths in patient:
  - ☐Accepts differences in others
  - ☐Asks for help when needed
  - ☐Courageous
  - ☐Curious
  - ☐Emotionally stable
  - ☐Establishes and keeps friendships
  - ☐Good listener
  - ☐Good sense of humor
  - ☐Intelligence
  - ☐Persistence
  - ☐Resilience
  - ☐Not Applicable (N/A) due to age
  - ☐Other: __________________________
- Client/caregiver-identified strengths in caregiver(s):
  - ☐Accepts differences in others
  - ☐Asks for help/support when needed
  - ☐Courageous
  - ☐Curious
  - ☐Maintains physical health
  - ☐Maintains mental health
  - ☐Establishes and keeps friendships/relationships
  - ☐Good listener
  - ☐Good sense of humor
  - ☐Intelligent
  - ☐Strong advocacy skills
  - ☐Resilience
  - ☐N/A (free text reason): ___________________________
  - ☐Other: ____
- Observed strengths (by social worker): ________________________
- Observed protective factors of family system (by social worker):___________________
  - ☐Access to community-based supports and resources
  - ☐Caregiver self-esteem

SOCIAL WORK BIOPSYCHOSOCIAL ASSESSMENT (Downtime)

- ☐ Family cohesion/functioning
- ☐ Adequate health literacy
- ☐ High level of parental/caregiver education
- ☐ Multiple-caregiver household
- ☐ Self-efficacy
- ☐ Stable access to food
- ☐ Stable employment
- ☐ Stable housing
- ☐ Stable access to transportation
- ☐ Strong caregiver/child relationship
- ☐ Strong support network
- ☐ N/A (state reason): ___________________________
- ☐ Other: _______________________________________

PATIENT'S PHYSICAL/MEDICAL CONDITION

Chief compliant: ____
Secondary diagnoses: ____
Physical/cognitive ability/disability: _____________________________________
Method of communication (for patient):

- ☐ Verbal
- ☐ Nonverbal
- ☐ Use of augmentative communication
- ☐ Sign language
- ☐ Gestures/facial expressions

CAREGIVER(S) PHYSICAL AND EMOTIONAL WELL-BEING

Current coping with hospitalization: ___________________________________
Processing/coping with patient diagnosis: ______________________________
Underlying mental health concerns: ___________________________________
Underlying physical health concerns: _________________________________
Barriers to caregiver efficacy/ ability to provide care to patient: __________________

FAMILY SYSTEM BARRIERS (Drop Down)

Financial

- ☐ Loss of employment
- ☐ Reduced hours
- ☐ Underemployment
- ☐ Loss of Benefits [Social Security (SS), SSI, SNAP, Medicaid, etc.]
- ☐ Ineligible for benefits
- ☐ Other: _____________________________________

SOCIAL WORK BIOPSYCHOSOCIAL ASSESSMENT (Downtime)

Food insecurity

- ☐ Running out of food
- ☐ Not enough money to purchase food
- ☐ Not enough money to purchase "healthy food"
- ☐ Re-directing money for food to bills/rent
- ☐ Difficulty purchasing/obtaining food while child in the hospital
- ☐ Other: ___________________________________

Health Literacy Concerns

- ☐ Related to understanding of specific medical condition
- ☐ Related to self-management of medical condition once patient is home
- ☐ Other: ____

Mental/behavioral health concerns

- ☐ In need of outpatient mental health services
- ☐ Community lacks mental health resources that the patient/family needs [Applied Behavioral Analysis (ABA), Functional Family Therapy (FFT), etc.]
- ☐ Prefers in-person mental health services, limited availability
- ☐ Does not engage/limited engagement with virtual mental health services
- ☐ Unable to secure a pediatric psychiatrist
- ☐ On wait list for mental health services
- ☐ Substance use
- ☐ None reported by family
- ☐ None reported by family. However, medical team/SW observes needs in this area.
- ☐ Other: ____

Safety

- ☐ Crib safety (Does the patient have a safe space to sleep?)
- ☐ Gun safety
- ☐ Over-the-Counter (OTC) and prescription medication secured
- ☐ Abuse/neglect
- ☐ Domestic violence
- ☐ Elopement concerns
- ☐ Human/sex trafficking
- ☐ Transportation or car seat safety
- ☐ Home/community environment: ____________________________ (explain)
- ☐ Other: ____

School concerns

- ☐ IEP/Special Education concerns
- ☐ Bullying
- ☐ Truancy
- ☐ Medical support (nursing) needed in school
- ☐ 504 plan needed
- ☐ Home school concerns
- ☐ School performance concern/School evaluation needed
- ☐ Other: ____

SOCIAL WORK BIOPSYCHOSOCIAL ASSESSMENT (Downtime)

Communication barriers (for caregivers):

- ☐Speech delay [Currently followed by Speech-language pathologist (SLP), No SLP]
- ☐American Sign Language (ASL) only
- ☐Language Spoken at home (Spanish, Dialect)
- ☐Nonverbal

Housing

- ☐Housing insecure
- ☐Living in a motel
- ☐In foreclosure
- ☐Homeless
- ☐Shelter
  - Name of shelter and address: ___________________________
- ☐Unable to afford rent/mortgage
- ☐Owes back rent/mortgage payments
- ☐Environmental concerns in the home (mold, black mold, water damage, pests, other)
- ☐Other: ____

Accommodations

- ☐RMH room request
- ☐Sleep room request
- ☐Ineligible for RMH
- ☐Other: ____

Adherence to medical plan

- ☐Family perspective on medical plan:
  - ☐Effective/meeting the patient's needs
  - ☐Not effective/not meeting the patient's needs
- ☐Family identified concerns with the medical plan: ________________
- ☐Family identified needs for the medical plan: __________________________
- ☐Barriers to family/provider collaboration: ______________________________
- ☐Provider concerns with adherence to medical plan: ______________________

Transportation

- ☐Medical Assistance Transportation (e.g. Motivcare)
- ☐Circulation
- ☐Gas Card
- ☐Other: _______________________________________

NEMOURS
CHILDREN'S HEALTH

Patient Name:
MRN:
DOB:

SOCIAL WORK BIOPSYCHOSOCIAL ASSESSMENT (Downtime)

COMMENTS

____
____
____
____
____
____
____
____
____
____

CARE PLAN

____
____
____
____
____

SOCIAL WORK DISPOSITION

☐ No further intervention warranted
☐ Social work will continue intervention

Social worker provided contact information to family: ☐ Yes ☐ No

Signature: ________________________________ Date: ______________ / Time: _____

Printed name: _____________________________ Phone number: ________________

Legend: ED: Emergency Department; SW: Social Work; PT: Physical Therapy; OT: Occupational Therapy; SSI: Supplemental Security Income; TANF: Temporary Assistance for Needy Families; SNAP: Supplemental Nutrition Assistance Program; WIC: Women, Infants and Children; MH: Mental Health; IDD: Intellectual Developmental Disabilities; Voc: Vocational; Rehab: Rehabilitation; IEP: Individualized Education Program ; N/A: Not Applicable; SS: Social Security; etc.: Et cetera; ABA: Applied Behavior Analysis; FFT: Functional Family Therapy; SLP: Speech-Language Pathologist; ASL: American Sign Language; RMH: Ronald McDonald House; e.g.: exempli gratia

*Nemours includes: The Nemours Foundation, a Florida not-for-profit corporation, its operating divisions and sites, and its affiliates and subsidiaries, including Nemours Children's Hospital, Delaware; Nemours Children's Hospital, Florida; Nemours Children's Hospital, Surgery Center, Bryn Mawr; and Nemours Children's Hospital, Surgery Center, Deptford; and all entities operating under the name Nemours Children's Health.
